# The Use of Water in the Dietary of Young Children

**Published:** 1883-11-20

**Authors:** Charles Remsen


### THE USE OF WATER IN THE DIETARY OF YOUNG CHILDREN.

By CHARLES REMSEN, M.D.

During my service as interne at the Nursery and Child's Hospital this summer, one factor in the production of infant mortality during hot weather has impressed itself strongly upon my notice. I refer to the ignorance shown by most persons, especially in the lower ranks of society, in regard to the amount of water required by children, either nursing or artificially fed.

Because the natural food of young children is in a fluid form, the laity generally suppose that no further supply of water is necessary. In medical writings on this topic, also, too little stress is laid on the importance of giving minute instructions in regard to this point.

When we consider the difference in the amount of fluids taken by an adult during hot and cool weather, it can readily be understood how a supply of water amply sufficient to meet all the requirements of a child in winter may be totally inadequate in the dry heats of summer. No provision for an increased supply, however, of this necessary principle is made through the natural channels; but, if the call for it be not attended to, the most serious consequences will result.

The blood having parted with much of its water to supply the increased evaportion from the surface of the body, will take up very quickly the fluid portion of any food introduced into the stomach, leaving the solids too thick to be easily digested; these then ferment and produce indigestion and colic, and, passing downward, diarrhœa, and hence a further drain upon the blood. As a consequence of the thickened state of the blood thus produced, the excretion of sweat is stopped and a condition of collapse and hyperpyrexia developed.

When we take into account the fact that there is a larger proportionate area from which evaporation takes place in children than in adults, and that there is a greater amount of activity displayed in all the vital processes in the former, it is evident that their punishment should be not only relatively greater in amount, but also given at much shorter intervals. In warm, dry weather babies will drink cool water every hour, or even at shorter intervals, if, as it should be, it is offered to them. When they are suffering from diarrhœa, the amount that they will drink and the improvement produced by it are astonishing.

The earliest sign showing the amount of water in the system to be below the normal standard is a slightly depressed condition of the anterior fontanelle. This may be present in children who otherwise present the appearance of perfect health ; yet any slight increase in temperature or deprivation of the breast for a few hours may give rise in them to a sudden hyperpyrexia. In nursing children, however, the attention is usually first aroused by fretfulness, a moderate rise of temperature and pulse, a hot, dry skin, and a constant desire to nurse. If a child in this state be given a free supply of water, and its nursing restricted in frequency, the symptoms will often disappear quickly and completely ; but, if not, collapse will soon come on.

In this condition the temperature ranges from 105° to 106° F., or higher ; the pulse is small and thready, and beats from 180 to 200 to the minute, the skin of the body feels painfully hot, while the extremities are cool ; the features are pinched and sunken, the eyes are half closed, with the pupils contracted ; the fontanelle is depressed, the hands are tightly shut ; the respiration is hurried and irregular, and consciousness seems abolished. In the majority of cases vomiting and diarrhœa have been absent, or, at most, the patient has had one or two small slimy stools. A child in this state will swallow water when offered to it with greediness and the expression of the utmost pleasure.

The treatment adopted at the Nursery for these emergencies consists in wrapping the patient in a wet sheet, applying cold to the head, and giving as much water by the mouth as the child will swallow.

The results of this simple method have been extremely satisfactory, the child becoming quiet, and even going to sleep, while all the threatening symptoms subsided with great rapidity.

The attention given to this point as a prophylactic measure among the inmates has been followed by a diminished rate of mortality, and a marked reduction in the number of gastric and intestinal complaints.

In conclusion, it may be said that if more care were directed toward giving children a proper amount of water, and restricting their hours of nursing or feeding, the mortality due to hot weather would decrease, and less would be heard about the troubles of teething.—*N. Y. Med. Rec.*

